# Efficient Surface Immobilization of Chemically Modified Hyaluronans for Enhanced Bioactivity and Survival of In Vitro-Cultured Embryonic Salivary Gland Mesenchymal Cells

**DOI:** 10.3390/polym13081216

**Published:** 2021-04-09

**Authors:** Sang-woo Lee, Junchul Kim, Xin Cong, Guang-Yan Yu, Ji Hyun Ryu, Kyungpyo Park

**Affiliations:** 1 Department of Physiology, School of Dentistry and Dental Research Institute, Seoul National University, Seoul 110-749, Korea; goodman23@snu.ac.kr (S.-w.L.); jckim1@snu.ac.kr (J.K.); 2 Department of Physiology and Pathophysiology, Peking University School of Basic Medical Sciences, Beijing 100191, China; congxin634@126.com; 3 Department of Oral and Maxillofacial Surgery, Peking University School and Hospital of Stomatology, Beijing 100081, China; gyyu@263.net; 4 Department of Carbon Convergence Engineering, Wonkwang University, Iksan, Joenbuk 54538, Korea

**Keywords:** hyaluronan, mesenchyme, feeder cell, catechol, CD44, branching morphogenesis, tissue engineering, salivary gland

## Abstract

Embryonic salivary gland mesenchyme (eSGM) secretes various growth factors (bioactives) that support the proper growth and differentiation of salivary gland epithelium. Therefore, eSGM cells can be used as feeder cells for in vitro-cultured artificial salivary gland if their survival and bioactivity are properly maintained. As eSGM is encapsulated in a hyaluronan (HA)-rich developmental milieu, we hypothesized that mimicking this environment in vitro via surface immobilization of HA might enhance survival and bioactivity of eSGM. In this study, various HA derivatives, conjugated with catechol (HA–CA), thiol (HA–SH), or amine (HA–EDA) moieties, respectively, were screened for their efficacy of culturing eSGM-derived feeder cells in vitro. Among these HA derivatives, HA–CA showed the highest surface coating efficiency and growth enhancement effect on the embryonic submandibular gland. In addition, the HA–CA coating enhanced the production of growth factors EGF and FGF7, but not FGF10. These effects were maintained when eSGM cells isolated from the embryonic salivary gland were re-seeded to develop the feeder layer cells. CD44s (a major HA receptor) in eSGM cells were clustered at the cell membrane, and enhanced EGF expression was detected only in CD44 cluster-positive cells, suggesting that membrane clustering of CD44 is the key mechanism for the increased expression of EGF.

## 1. Introduction

Hyaluronan (hyaluronic acid (HA)) is a natural biopolymer abundantly found in the extracellular matrix (ECM) of various human tissues. Due to its diverse bioactivities and excellent biocompatibility, HA has been widely studied for biomedical applications, including tissue engineering, drug delivery, regenerative medicine, and bio-lubrication [1]. However, natural HA has low mechanical strength and weak adhesiveness, which limit industrial applications of HA. To enhance the physicochemical properties of HA, various kinds of chemical modifications, including conjugation with the thiol (SH) [2], aldehyde [3], amine [4], methacrylate [5], and catechol (CA) moiety [6], have been suggested. In the various biomedical fields, chemically modified HAs have been used to enhance the bioactivity of adipose-derived stem cells [7], neural stem cells [6], endothelial cells [8], and salivary gland stem/progenitor cells [9]. Such biological effects of HA or HA derivatives are mediated by CD44, a receptor for HA ubiquitously expressed throughout various cell types [10]. CD44s can interact with other membrane-bound receptors or soluble factors, and multiple CD44s can cluster together to send different cell signals [11,12].

It has been widely accepted that the replacement of damaged salivary glands with in vitro-generated artificial salivary glands is the fundamental strategy for radiation-induced hyposalivation [13]. However, the salivary gland consists of diverse cell types and a complex branching structure, so mimicking the developmental process of the embryonic salivary gland is considered the most reasonable approach for salivary gland tissue engineering [14]. To study the salivary gland developmental process, mouse embryonic submandibular glands (eSMGs) cultured in vitro at an air–media interface have been used as a robust study model for several decades [15,16]. At embryonic day (E) 12, eSMG cells have only a single epithelial bud surrounded by mesenchyme. Due to the continuous cross-talks between the epithelium and mesenchyme, however, the epithelial buds dynamically proliferate, divide, and elongate to form hundreds of epithelial buds and branching structures up to E16~18 [16]. This dynamic process is called “branching morphogenesis.” During this process, the mesenchyme acts as a feeder layer that nourishes and supports stem/progenitor cells in epithelial buds by secreting soluble growth factors (e.g., fibroblast growth factors FGF7 and FGF10, and epidermal growth factor (EGF)) and providing them with ECM components (e.g., collagen, heparin sulfate, laminin, and HA) [16]. It has also been reported that co-culture of separated embryonic mesenchyme and salivary gland organoids markedly boosts expression levels of salivary gland-specific functional proteins in the salivary gland organoids [17]. Therefore, maintaining in vivo bioactivity or niche factor levels in vitro is crucial for efficient in vitro generation of artificial salivary glands. We previously found thick layers of HA surrounding and directly contacting the mesenchyme of eSMGs within the developmental milieu [9]. Therefore, we hypothesized that mimicking such an HA-rich developmental milieu on the culture surface in vitro may support the efficient formation of a highly bioactive mesenchymal feeder layer. Mimicking the physicochemical properties of the surrounding niche has been considered the most effective method to regulate the bioactivities of stem cells in vitro. For example, osteogenic differentiation of oral tissue-derived stem cells can be regulated by mechanical stiffness and ECM components of the niche [18,19].

In the present study, we synthesized various HA derivatives by conjugation with the catechol (CA) (HA–CA), thiol (SH) (HA–SH), and amine (HA–ethylenediamine (EDA)) moiety, respectively, for efficient coating on the embryonic salivary gland mesenchyme (eSGM) culture platform. Among the three HA derivatives, HA–CA showed the highest surface coating efficiency and eSMG growth enhancement effect by boosting mesenchymal growth factor production. A mechanistic investigation revealed that HA–CA coating-induced membrane clustering of mesenchymal CD44 is the key initiator for the boosted expression of mesenchymal growth factors.

## 2. Materials and Methods

### 2.1. Materials

HA (200 kDa) was purchased from Lifecore Biomedical, Inc. (Chaska, MN, USA) and 1-ethyl-3-(3-dimethylaminopropyl)-carbodiimide hydrochloride (EDC) was purchased from TCI (Tokyo, Japan). EDA (dihydrochloride salt), cysteamine hydrochloride, and dopamine hydrochloride were purchased from Sigma-Aldrich (St. Louis, MO, USA). All other chemicals were of analytical grade.

### 2.2. Synthesis of Amine-Functionalized HA (HA–Amine; HA–EDA)

EDA-modified HA (HA–amine) was synthesized according to a previous report with slight modifications [20]. In brief, HA (500 mg) was dissolved in distilled deionized water (DDW, 50 mL; final concentration = 10 mg/mL). After adding EDC (380 mg) and EDA (1.32 g), the solution was reacted for 12 h, and the pH of the reaction solution was adjusted to about 6.5. The product was dialyzed (SpectraPor 3.5 kDa molecular weight cutoff (MWCO); Spectrum Laboratories, Rancho Dominguez, CA, USA) against DDW for 3 days and lyophilized. The synthesis of HA–amine was confirmed by ^1^H NMR spectroscopy (500 MHz NMR; Bruker, Billerica, MA, USA), as previously reported [21].

### 2.3. Synthesis of Thiolated HA (HA–Thiol; HA–SH)

Thiol groups were introduced into HA by standard EDC chemistry. Briefly, EDC (380 mg) and 1-hydroxybenzotriazole (284 mg) were added to a solution of HA (500 mg) in 50 mL DDW (final concentration = 10 mg/mL). After 30 min, cysteamine hydrochloride (284 mg) was added to the reaction solution. The reaction was allowed to proceed for 12 h, and the pH was adjusted to about 6.5. The synthesized HA–SH was purified by dialysis (SpectraPor 3.5 kDa MWCO; Spectrum Laboratories) and lyophilized. The synthesis and chemical structures of HA–thiol were determined by ^1^H NMR spectroscopy (500 MHz NMR; Bruker), as previously reported [22].

### 2.4. Synthesis of CA-Functionalized HA (HA–Catechol; HA–CA)

Conjugation of CA moieties with HA (HA–catechol) was performed according to our previous report [9]. Briefly, EDC (474 mg) and dopamine hydrochloride (388 mg) were added to a solution of HA (1 g) in 100 mL DDW (final concentration = 10 mg/mL) and reacted for 12 h. The pH of the reaction solution was adjusted to 5.5 using 1 M HCl. The product was dialyzed (SpectraPor 3.5 kDa MWCO; Spectrum Laboratories) against a HCl solution (pH 5.0) for 2 days and then against deionized water for 6 h and lyophilized. The catechol conjugation into the HA backbone was confirmed by a UV–Vis spectrophotometer (UV-1900i; Shimadzu, Kyoto, Japan), as previously described [9,23].

### 2.5. HA Derivative Coating on Polycarbonate Membrane

Each HA derivative was dissolved in Dulbecco’s modified Eagle medium/Nutrient Mixture F12 (DMEM/F12, Gibco, Grand Island, NY, USA) culture medium to a final concentration of 2 mg/mL, and then polycarbonate (PC) membranes were dipped in the solution at 37 °C for 24 h. After the incubation, PC membranes were gently washed with DDW and dried under a nitrogen atmosphere.

### 2.6. Characterization of Surfaces Coated with HA Derivatives

For Alcian Blue staining, PC membranes coated with various HA derivatives were immersed in Alcian Blue solution (Sigma-Aldrich, B8438) for 5 min and gently washed with PBS three times. The PC membranes were then dried and imaged under a stereomicroscope (Leica S6D; Leica Microsystems GmbH, Wetzlar, Germany).

### 2.7. Isolation and In Vitro Culture of Mouse eSMG

eSMGs were extracted from mouse embryos at E12–14. Isolated eSMGs were placed on the bright side of a porous PC filter membrane (Sigma-Aldrich, 110405) floating on DMEM/F12 (1:1; Gibco) supplemented with 150 μg/mL ascorbic acid (Sigma-Aldrich, A5960), 50 μg/mL transferrin (Sigma-Aldrich, T8158), and 1% (*v*/*v*) penicillin–streptomycin (Gibco, 15140122). The eSMGs were cultured in an incubator for 48 h (37 °C, 5% CO_2_). The animal experiment protocol used in this study was approved by the Seoul National University Institutional Animal Care and Use Committee (approval number: SNU-190320-7).

### 2.8. mRNA Isolation and Quantitative Real-Time RT-PCR (qRT-PCR)

Approximately 8~12 eSMGs were collected per experimental group, and dissolved in TRIzol reagent (Thermo Fisher Scientific, 15596018; Waltham, MA, USA) by SuperFast Prep-2 (MP Biomedicals, MP116012500; Santa Ana, CA, USA). Total RNA was extracted using the Direct-Zol RNA MicroPrep Kit (Zymo Research, R2060; Tustin, CA, USA). The isolated total RNAs were converted to cDNA, and qRT-PCR was performed with 35 cycles of denaturation at 94 °C for 40 s, annealing at 55 °C for 40 s, and extension at 72 °C for 40 s, with a final extension at 72 °C for 10 min. Primer sequences for mouse EGF, FGF7, FGF10, and GAPDH were as follows: mouse EGF, forward 5′-TACTCAGCGTCACAGCATGG-3′ and reverse 5′-AGCCACCCTCATAATCACAG-3′; mouse FGF7, forward 5′-AATGACATGAGTCCGGAGCAA-3′ and reverse 5′-CCATAGGAAGAAAATGGGCTG-3′; mouse FGF10, forward 5′-GGATACTGACACATTGTGCCTCAG-3′ and reverse 5′-TGTTTTTTGTCCTCTCCTGGGAGC-3′.

### 2.9. Separation of Mesenchyme and Formation of Mesenchymal Feeder Cell Layer

Briefly, approximately 10–15 eSMGs were incubated with 0.5 U/mL Dispase-I (Life Technologies, 17105-041; Carlsbad, CA, USA) dissolved in calcium/magnesium-free Hank’s balanced salt solution for 20 min, and washed twice with 3% bovine serum albumin (Sigma-Aldrich, A2934-25G) dissolved in DMEM/F12 (1:1) (Gibco, 14175-095). Mesenchyme and epithelium of eSMGs were then separated under a stereomicroscope using ultrafine forceps made of Dumostar alloy (Dumont #5 forceps, 0.05 mm × 0.01 mm; Fine Science Tools, 11295-20; Foster City, CA, USA). The isolated mesenchyme was immersed in RPMI medium containing 1 mg/mL collagenase 4 (Sigma-Aldrich, C5138-100MG), and then incubated under continuous rotation using a MACSmix Tube Rotator (Miltenyi Biotec, 130-090-753; Bergisch Gladbach, Germany) at 37 °C for 20 min. Enzymatically digested mesenchyme was mechanically dissociated using a gentleMACS Dissociator (Miltenyi Biotec, 130-093-235) operated in “hTumor 3” mode. Dissociated mesenchymal cells were washed three times with DMEM/F12 (1:1) (Gibco). After counting the cells, 5 μL of dissociated eSMG cells (10,000 cells) were placed on the floating PC membrane to be cultured in an incubator for 48 h (37 °C, 5% CO_2_). Forty-eight hours after the seeding, the viability test was performed. For this, 10 µL of Cell Counting Kit-8 (CCK-8; Dojindo, CK04-11; Rockville, MD, USA) solution were added to the culture media of each experimental group and incubated in an incubator for 1 h. Afterward, 100 µL of media were drawn to measure absorbance at 450 nm with a plate reader (BioTek, Synergy 2; Winooski, VT, USA).

### 2.10. Immunofluorescence Staining

Cultured eSMGs or dissociated mesenchymal cells were fixed with 4% paraformaldehyde at room temperature (RT) for 18 min, washed twice with phosphate-buffered saline (PBS), and permeabilized with PBS containing 0.5% (*v*/*v*) Triton-X100 (PBSX) for another 20 min at RT. The permeabilized eSMGs or mesenchymal cells were then blocked by immersion in PBSX containing 10% normal donkey serum (NDS; Sigma-Aldrich) and 1% mouse-on-mouse (MOM) blocking reagent (Vector Laboratories, MKB-2213-1; Burlingame, CA, USA) at RT for 3 h. After the blocking step, eSMGs or mesenchymal cells were incubated with PBSX containing primary antibodies (1:100) and 3% NDS at 4 °C for 12 h. The primary antibodies used in the procedure were as follows: goat polyclonal anti-c-kit antibody (R&D System, AF1356; Minneapolis, MN, USA), rat monoclonal anti-CD44 antibody (Abcam, ab157107; Cambridge, UK), mouse monoclonal anti-TUJ1 (R&D System, MAB1195), rabbit polyclonal anti-E-cadherin (Cell Signaling Technology, 3195S; Beverly, MA, USA), mouse monoclonal anti-EGF (Invitrogen, MA5-15606), and rabbit polyclonal anti-FGF7 (Invitrogen, PA5-49715; Carlsbad, CA, USA). To remove unbound primary antibodies, the cells were washed four times with PBSX (10 min per wash) at RT. Afterward, eSMGs or mesenchymal cells were incubated with PBSX–3% NDS solution containing secondary antibodies (1:250) and DAPI (1:1000) at 4 °C for 12 h. The secondary antibodies used in this step were as follows: donkey anti-goat IgG (H + L) Alexa Fluor^®^ 488 conjugate (Abcam, ab150129), donkey anti-rabbit IgG (H + L) Alexa Fluor^®^ 594 conjugate (Abcam, ab150080), donkey anti-mouse IgG (H + L) Alexa Fluor^®^ 594 conjugate (Invitrogen, A21203), and donkey anti-rat IgG (H + L) Alexa Fluor^®^ 647 conjugate (Abcam, ab150167). To remove unbound secondary antibodies, the cells were washed four times with PBSX (10 min per wash). Finally, eSMGs or mesenchymal cells attached to the filter paper were mounted on a glass slide for imaging by confocal laser scanning microscopy (CLSM).

### 2.11. Imaging of In Vitro-Cultured Mouse eSMGs

All bright-field images were obtained using a digital inverted fluorescence microscope (Nikon Ti; Nikon, Tokyo, Japan). Immunofluorescence staining images were obtained using a Carl Zeiss LSM700 confocal microscope equipped with 10×, 20×, and 40× Plan-Apochromat lenses (Carl Zeiss, Jena, Germany). For multi-color imaging, 405, 488, 555, and 647 nm excitation lasers were used. For each image, around 3–4 *z*-sections were obtained and merged into one image via the maximum intensity projection tool in Zen 2010 Blue software (Carl Zeiss). For bud counting, epithelial end bud numbers were manually counted based on bright-field images. For epithelial area calculation, the E-cadherin (epithelial cell marker)-positive area was measured by Zen 2010 Blue software (Carl Zeiss). For bud/duct ratio calculation, the bud area (the c-Kit-positive/E-cadherin-positive area) and duct area (E-cadherin-positive/c-Kit-negative area) were calculated by Zen 2010 Blue software (Carl Zeiss). Epithelial bud size was measured as the manually defined circular area of E-cadherin-positive cell aggregates in Zen 2010 Blue software (Carl Zeiss).

### 2.12. Data Analysis

The Shapiro–Wilk test (α = 0.05; *n* ≥ 3 replicates per group) was performed to test normality, and the modified Levene’s test (α = 0.05) was performed on all data sets with *n* ≥ 3 replicates to test the homogeneity of variances. Data sets that passed both tests were subjected to one-way ANOVA, followed by Dunnett’s test. Those that did not pass were subjected to non-parametric ANOVA (Kruskal–Wallis ANOVA), followed by non-parametric Dunnett’s test in Prism 8.1.0 software (GraphPad, La Jolla, CA, USA). Significance levels were assigned as follows: * *p* < 0.05, ** *p* < 0.01, and **** *p* < 0.001.

## 3. Results and Discussion

### 3.1. Synthesis of HA Derivatives and Their Coating Application

**Figure 1:**
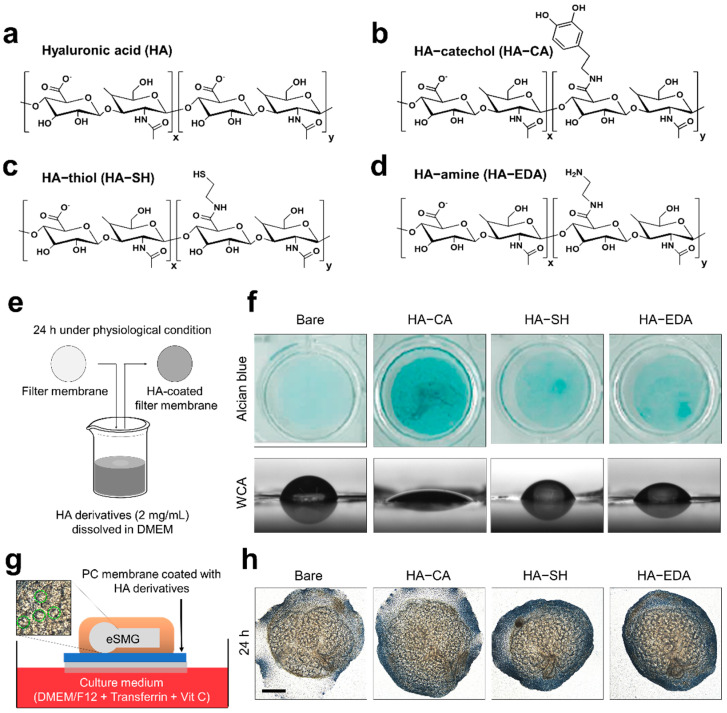
Schematic chemistries of (**a**) HA, (**b**) HA–catechol, (**c**) HA–thiol, and (**d**) HA–amine are depicted. (**e**) Schematic diagram of polycarbonate (PC) membrane coated with HA derivatives. (**f**) Evaluation of coating efficiencies of HA derivatives on the polycarbonate (PC) membranes by Alcian Blue staining (upper panel) (*n* = 3) and water contact angle (WCA) analysis (lower panel) (*n* = 3). (**g**) Schematic diagram of in vitro culture of embryonic submandibular glands (eSMGs). Epithelial buds are indicated with green dotted circles. (**h**) Bright-field images of E14 eSMGs cultured on PC membranes coated with HA derivatives for 48 h. Scale bar = 200 µm (*n* = 3).

To determine the effects of various functionalized HA derivatives as surface coating materials, we synthesized and tested the HA–amine (HA–EDA), HA–thiol (HA–SH), and HA–catechol (HA–CA) conjugates. Figure 1a–d show the chemical structures of HA and the three conjugates. Previously, HA–SH has been used as a scaffold for three-dimensional culture of primary salivary gland cells [24,25], and HA–CA has been used to surface-immobilize HA on an embryonic salivary gland culture platform [9]. HA–thiol and HA–CA were also synthesized by standard carbodiimide chemistry using cysteamine and dopamine, respectively. Although HA–EDA has not yet been used for salivary gland tissue engineering, it was selected as a candidate in the current study because the amine moiety can interact with various biological molecules [26,27]. To synthesize HA–amine, the carboxylic acid groups in HA were activated by EDC and further reacted with the amino groups in EDA to form amide bonds. Excess EDA was added to the reaction solution to prevent undesirable crosslinking reactions between HA molecules. The estimated degree of catechol, thiol, and ethylene diamine substitution was 7.3%, 24.3%, and 15.2%, respectively.

Next, we tested the coating efficiency of the HA derivatives. To prepare the HA–derivative-coated PC membranes, the HA–amine, HA–thiol, and HA–catechol conjugates were dissolved at 2 mg/mL in DMEM/F12 culture media. After complete dissolution of the HA derivatives, the PC membranes were placed in the solutions of the conjugated HAs at 37 °C for 24 h. After the PC membranes were coated with conjugated HA, the surfaces were vigorously washed with PBS solution and DDW, and dried under a nitrogen atmosphere (Figure 1e) before evaluation of coating efficiency by the Alcian Blue assay and water contact angle (WCA) assay. Alcian Blue is a cationic dye that forms blue-colored insoluble complexes with glycosaminoglycan [28], allowing visualization of HAs bound to the PC membranes. Among the three HA derivatives, only the HA–CA-coated membrane showed a deep blue color and thus exhibited satisfactory efficiency for surface immobilization of HA (Figure 1f, upper panel). The other groups displayed a pale blue color. Similarly, in the WCA assay, only the HA–CA-coated PC membrane (WCA = 23.5°) exhibited hydrophilic properties while PC membranes coated with either HA–SH (WCA = 43.5°) or HA–EDA (WCA = 40.8°) showed hydrophobic properties similar to bare PC membrane (WCA = 62°) (Figure 1f, lower panel).

To evaluate the bioactivity of surfaces coated with HA derivatives, E14 eSMGs were placed on the PC membranes coated with HA derivatives and cultured for 48 h (Figure 1g). Classically, eSMGs can be in vitro-cultured at the air–media interface; after isolation from the mandibles of mouse fetus, eSMGs are placed on a porous PC filter membrane floating on serum-free DMEM/F12 media supplemented with ascorbic acid and transferrin [15,16]. As the eSMG grows and matures, its size and bud number exponentially increase through branching morphogenesis. Therefore, the size and bud number of eSMGs are used as criteria for evaluating the degenerative or enhancement effects of biomaterials. Epithelial buds are indicated with green dotted lines in Figure 1g. eSMGs showed significantly increased size and bud number when cultured on the HA–CA-coated membrane and no significant enhancement when cultured on the HA–SH- or HA–EDA-coated membrane compared to the bare membrane group (Figure 1h and Figure S1). These results indicated that the amount of immobilized HA was crucial for the growth enhancement of eSMGs cultured in vitro. Among the three HA derivatives, only HA–CA achieved a coating efficiency sufficient to exert such biological enhancement effects.

### 3.2. Biocompatibility of HA Derivatives for Embryonic Salivary Gland Culture

**Figure 2:**
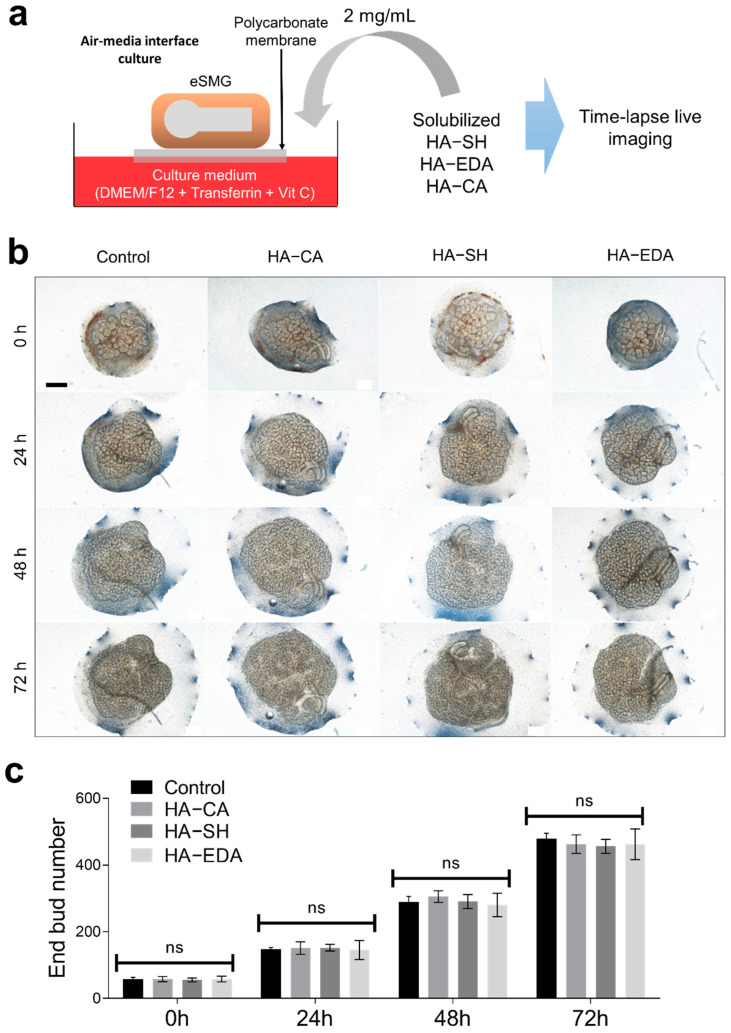
(**a**) Schematic diagram of cytotoxicity assay using eSMG culture model. (**b**) Time-lapse bright-field imaging of E14 eSMGs cultured with solubilized HA derivatives. Scale bar = 200 µm (*n* = 3). (**c**) Quantification of bud number at each time point (*n* = 3). Data are expressed as average ± SEM (Figure 2c). Non-significant (ns; *p* > 0.05) by one-way ANOVA with Dunnett’s tests (within each time point).

To confirm whether the absence of growth enhancement effect on HA–SH and HA–EDA is due to their innate toxicity, we tested the cytotoxicity of the synthesized HA derivatives. Solubilized HA derivatives were introduced to the in vitro culture system of eSMGs. For the cytotoxicity assay, E14 eSMGs were cultured for up to 72 h with culture media supplemented with solubilized HA derivatives (final concentration of 2 mg/mL), and their morphologies were monitored every 24 h (Figure 2a). If HA derivatives are toxic to eSMGs, there will be a morphological anomaly or decreased bud number. No significant morphological defect was observed in all HA derivative groups, indicating that all HA derivatives are biocompatible for salivary gland tissue engineering (Figure 2b,c).

### 3.3. HA–CA Coating Specifically Enhances Proliferation of Epithelial Buds, But Not Ducts

**Figure 3:**
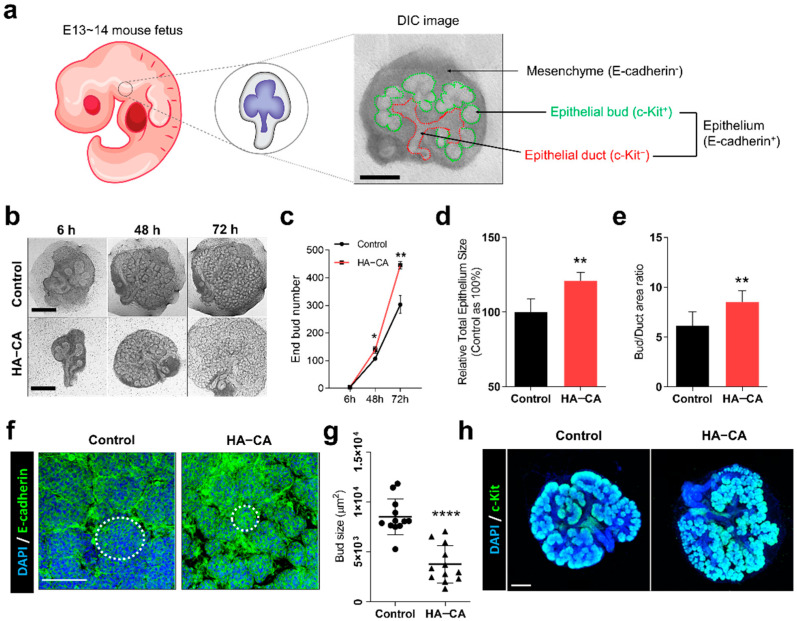
(**a**) Schematic diagram of E13~14 eSMG anatomy. Mesenchyme (E-cadherin negative) and epithelium (E-cadherin positive) are indicated, and within an epithelium, epithelial bud (c-Kit positive) and epithelial duct (c-Kit negative) structures are indicated in the differential interference contrast (DIC) image of E13.5 eSMG. (**b**) Time-lapse bright-field images of E13 eSMGs cultured on either bare (control) or HA–CA-coated surface. Scale bar = 200 µm (*n* = 4). (**c**) Time-dependent end bud number of eSMGs cultured on either control or HA–CA-coated surface (*n* = 4). (**d**) Total epithelium sizes of the control and group HA–CA are measured by ImageJ and expressed as a relative percentage (control as 100%). (**e**) Bud/duct area ratios of the control and group HA–CA are quantified by ImageJ (*n* = 4). (**f**) Immunostaining images of epithelial buds (E-cadherin in green and DAPI in blue) of eSMGs cultured on control and HA–CA-coated surfaces for 48 h. Dotted circles show the size of buds in each group. Scale bar = 50 µm (*n* = 3). (**g**) Quantification of bud size based on the immunostaining images of epithelial buds in control and HA–CA group (*n* = 12). (**h**) Immunostaining images of epithelial stem/progenitor cells (c-Kit in green and DAPI in blue) in E13 eSMGs cultured on control and HA–CA-coated surfaces for 48 h. Scale bar = 200 µm (*n* = 3). Data are expressed as average ± SEM (Figure 3c–g). ** *p* < 0.01, **** *p* < 0.001, ns = non-significant (*p* > 0.05) by unpaired *t*-test.

To further elucidate the mechanism of the HA–CA coating-mediated enhancement in eSMG growth, the characteristics of eSMGs grown on HA–CA-coated surfaces were examined. To examine the effects of HA–CA coating on comparatively more undifferentiated and primitive cells in eSMGs, we used E13 rather than E14 eSMGs. The anatomy of E13 eSMGs is briefly described in Figure 3a. In E13 eSMGs, epithelium, expressing E-cadherin, is encapsulated by mesenchyme which does not express E-cadherin (Figure 3a). The epithelial structure can be divided into epithelial buds (expressing c-Kit stem/progenitor cell marker; becoming saliva-secreting functional acini when fully matured) and epithelial duct (not expressing c-Kit; becoming luminal duct for saliva outflow when fully matured) (Figure 3a). Time-lapse tracking of eSMG morphology showed a significant bud number difference between the control and HA–CA group at 48 h after culture, and a further increase at 72 h (Figure 3b,c). Similarly, the total epithelium size of eSMGs grown on the HA–CA-coated surface was increased by 24% (Figure 3d). Interestingly, within an epithelium, a significantly increased bud/duct ratio was observed in the HA–CA group, indicating that HA–CA enhanced the proliferation of epithelial bud cells preferentially over duct cells (Figure 3e). CLSM images of the epithelial bud region revealed a significantly smaller average bud size in the HA–CA group than in the control group (Figure 3f,g). These results suggest that stem cells in the epithelial buds become highly proliferative, promoting a relatively faster clefting/budding process. As we expected, higher expression of c-Kit, a highly proliferative epithelial bud stem/progenitor cell marker [29,30], was observed in eSMGs grown on the HA–CA-coated surface than on the bare membrane (Figure 3h).

### 3.4. Immobilized HA–CA, Not Solubilized Form, Boosts EGF and FGF7 Production from eSMG Mesenchyme

**Figure 4:**
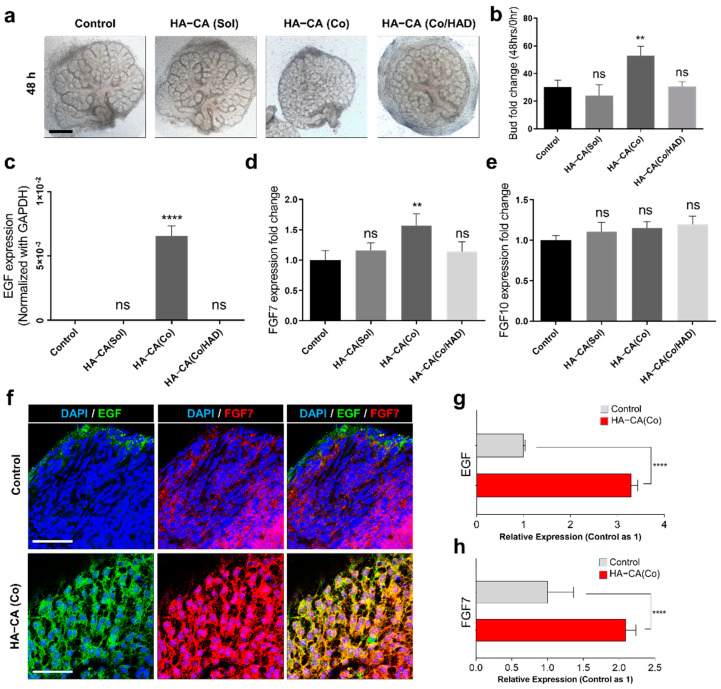
(**a**) Bright-field images of E13 eSMGs cultured for 48 h in control, solubilized HA–CA (HA–CA_(Sol)_), HA–CA-coated surfaces (HA–CA_(Co)_), and hyaluronidase-treated HA–CA-coated surfaces (HA–CA_(Co/HAD)_). Scale bar = 200 µm (*n* = 4). (**b**) Bud number fold changes (48 h/0 h) of eSMGs cultured in each group (*n* = 4). (**c**–**e**) mRNA expression level of (**c**) EGF, (**d**) FGF7, and (**e**) FGF10 in eSMGs cultured for 48 h under each condition (*n* = 3). (**f**) Immunofluorescence images of mesenchymal EGF (green) and FGF7 (red) expression in eSMGs cultured for 48 h on control and HA–CA-coated surface. DAPI (blue). Scale bar = 50 µm (*n* = 3). Quantification of mesenchymal (**g**) EGF and (**h**) FGF7 expression based on the immunofluorescence images. Control (gray) and HA–CA_(Co)_ (red) (*n* = 4). Data are expressed as average ± SEM (Figure 4b–h). ** *p* < 0.01, **** *p* < 0.001, ns = non-significant (*p* > 0.05) by unpaired *t*-test (Figure 4g,h) and one-way ANOVA with Dunnett’s test (Figure 4b–e).

Based on the previous results, we hypothesized that HA–CA enhances the production of soluble mesenchymal factors that specifically enhance the proliferation and clefting/budding process of epithelial buds. It has been well characterized that EGF, FGF7, and FGF10 are responsible for epithelial bud proliferation [31], epithelial clefting/budding [32,33], and duct elongation [32,33], respectively. To prove our hypothesis, we determined the mRNA expression levels of EGF, FGF7, and FGF10 under various conditions: control, solubilized HA–CA supplemented to culture media (HA–CA_(Sol)_), HA–CA coating (HA–CA_(Co)_), and hyaluronidase (HAD)-treated HA–CA-coated surface (HA–CA_(Co/HAD)_). Interestingly, the growth enhancement effect was not observed in the HA–CA_(Sol)_ group, suggesting that the presentation form of HA–CA is crucial for the bioactivity (Figure 4a,b). Furthermore, as anticipated, when immobilized HA–CA was removed by HAD treatment, the enhancement effect disappeared (Figure 4a,b), with a similar trend observed in the mRNA expression levels of EGF and FGF. Surprisingly, the mRNA expression level of EGF in the HA–CA_(Co)_ group was approximately 1000 times higher than in the other groups, but no such effect was exerted by HA–CA_(Sol)_ (Figure 4c). In addition, only the HA–CA_(Co)_ group showed a significant increase (1.57 times higher) in the mRNA expression level of FGF7 compared with the control group (Figure 4d). Again, the HA–CA_(Sol)_ group displayed no such enhancement effect (Figure 4d). Lastly, there was no significant increase or decrease in the mRNA expression level of FGF10, which supported the high bud/duct ratio observed in the previous result (Figure 4e). To confirm the protein expression level and localization of EGF and FGF7, immunofluorescence staining was performed. EGF was barely expressed and FGF was moderately expressed in the mesenchyme of the eSMGs in the control group, but both growth factors were robustly expressed in the mesenchyme of eSMGs in the HA–CA_(Co)_ group (Figure 4f,g).

### 3.5. HA–CA-Coated Surface Supports Viability and Bioactivity of Isolated eSMG Mesenchyme by inducing CD44 Clustering

**Figure 5:**
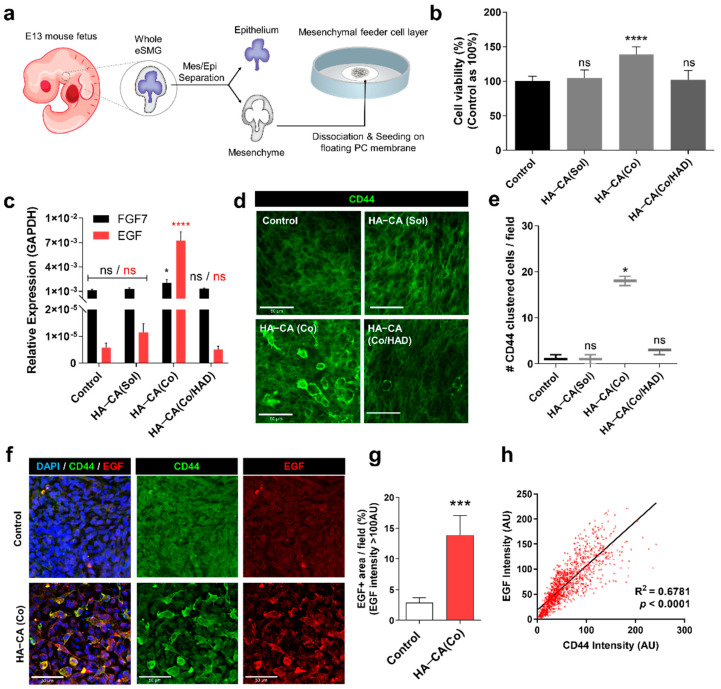
(**a**) Schematic diagram of embryonic salivary gland mesenchymal (eSGM) feeder cell layer formation. (**b**) Cell viability of eSGM cells cultured for 48 h under each condition (*n* = 6). (**c**) mRNA expression level of EGF and FGF7 in eSGM cells cultured for 48 h under each condition (*n* = 3). (**d**) Immunofluorescence images of mesenchymal CD44s at 4 h after seeding. Scale bar = 50 µm (*n* = 3). (**e**) Quantification of CD44-clustered eSGM cells within a defined visual field area (*n* = 3). (**f**) Immunofluorescence images of mesenchymal CD44s and EGF in eSGM cells cultured for 48 h on control or HA–CA-coated surfaces. Scale bar = 50 µm (*n* = 3). (**g**) Quantification of mesenchymal EGF expression in eSGM cells. Pixel area where EGF intensity is higher than 100 AU are measured and divided by the defined visual field area (*n* = 4). (**h**) Linear correlation is plotted between signal intensities of CD44 and EGF in the immunofluorescence images. R^2^ and *p*-values are noted in the graph. Data are expressed as average ± SEM (Figure 5b,c,g) or median with interquartile range (Figure 5e). * *p* < 0.05, *** *p* < 0.005, **** *p* < 0.001, ns = non-significant (*p* > 0.05) by unpaired *t*-test (Figure 5g), one-way ANOVA with Dunnett’s tests (Figure 5b,c), and Kruskal–Wallis ANOVA with non-parametric Dunnett’s test (Figure 5e).

As mentioned above, mesenchymal cells from embryonic salivary glands have a great potential to be used as feeder cells for in vitro generation of artificial salivary glands in the future. Hosseini et al. reported that as a feeder cell, mesenchymal cells from embryonic salivary glands are much more bioactive than NIH3T3 cells or bone marrow-derived mesenchymal stem cells [17]. However, due to the continuous interaction of the eSMG mesenchyme with the epithelium for its survival, separated mesenchyme becomes relatively unstable. Therefore, it is critical to confirm that the HA–CA coating is capable of supporting the growth and bioactivity of the eSGM without the presence of epithelium. To test this, we separated E13 eSMG mesenchyme from the epithelium and further dissociated mesenchyme into single cells. The dissociated embryonic mesenchymal cells were then seeded on either bare or HA–CA-coated PC membrane to create a feeder layer (Figure 5a). To test the proliferation rate of seeded mesenchymal cells, we performed the CCK8 assay. At 24 h after the seeding, mesenchymal cells on the HA–CA-coated surface showed 38% higher viability than the control group, indicating that the HA–CA coating enhances the proliferation rate of mesenchymal cells (Figure 5b). Next, to confirm whether the HA–CA coating could boost the mesenchymal feeder layer, qRT-PCR analyses of FGF7 and EGF were performed. FGF7 and EGF showed, respectively, approximately 1.8- and 1251-fold higher expression in the embryonic mesenchymal feeder layer grown on the HA–CA-coated surface than in the control group (Figure 5c). However, no enhancement effects were observed in the HA–CA_(Sol)_ or HA–CA_(Co/HAD)_ group, indicating that the immobilized HA–CA on the culture surface is the key factor (Figure 5c).

In this model, the mesenchyme is completely isolated from the epithelium, so a study of the mechanism of the HA–CA coating-induced effects on mesenchyme is more easily approachable. We hypothesized that the HA–CA coating-induced effects are mediated by CD44, abundantly expressed in mesenchyme from the early developmental stages (Figure S2). To examine the expression level and pattern of mesenchymal CD44 during the early phase of surface contact, we performed the CD44 immunostaining 4 h after the seeding. Surprisingly, approximately 30–40% of the mesenchymal cells on the HA–CA-coated surface showed membrane-localized and clustered expression of CD44s (Figure 5d,e). To confirm the correlation between CD44 clustering and the increased expression of EGF, we co-immunostained both CD44 and EGF in eSGM cells 24 h after the seeding. As could be expected, eSGM cells on the HA–CA-coated surface showed significantly enhanced expression of the EGF protein, and only CD44 clustering-positive cells showed increased expression of EGF (Figure 5f,g). These results explain the improved survival and proliferation rate of separated eSGM cultured on HA–CA-coated surfaces, as EGF has a self-stimulatory effect on eSGM cells [31]. The EGF signal intensity showed a significant linear correlation with the CD44 signal intensity (R^2^ = 0.6787, *p* < 0.0001), indicating that the CD44 clustering is the key mechanism for the HA–CA coating-mediated enhancement of growth factor production in eSGM (Figure 5h).

Although our HA-CA coating is only tested for a specific type of mesenchymal cells, eSGM cells, this coating platform can be used to regulate the proliferation, differentiation, and bioactivity of various oral tissue-derived stem cells, including dental pulp stem cells, periodontal ligament stem cells, dental follicle progenitors, and human periapical cyst–mesenchymal stem cells [34,35]. As these oral tissue-derived stem cells have mesenchymal stem cell-like properties and the ability to differentiate into various cell types such as hepatocytes, myocytes, nerves, adipocytes, and even hair follicle cells, the chemical conjugation of targeted substances (e.g., fibronectin, collagen, or bone morphogenetic protein-2) with adhesive catechol will provide an in vitro platform for efficient production of diverse regenerative cell types from oral tissue-derived stem cells [34,35].

## 4. Conclusions

In the present study, we discovered that HA–CA is the most optimized HA derivative for the surface immobilization of HA under physiological conditions. The growth enhancement effect on eSMGs was proportional to the amount of surface-immobilized HA. In detail, we found that the HA–CA coating preferentially enhances epithelial bud growth instead of duct growth. This phenomenon is due to the boosted production of growth factors specific to the bud growth—EGF and FGF7—from mesenchyme. In addition, both bioactivity and survival of separately cultured mesenchymal cells as a feeder cell layer are enhanced by the HA–CA coating. An investigation into the mechanism revealed that the immobilized form of HA–CA, but not the solubilized form, induces membrane localization and clustering of CD44 in mesenchymal cells. Furthermore, only CD44-clustered mesenchymal cells show enhanced expression of EGF. These results suggest that HA–CA coating should be widely applicable for the efficient in vitro generation of artificial salivary gland organoids.

## Data Availability

All relevant data are within the paper and its Supplementary Materials.

## Supplementary Materials

The following are available online at https://www.mdpi.com/article/10.3390/polym13081216/s1, Figure S1: Quantification of bud number 24 h after eSMG culture on bare, HA–CA-coated, HA–SH-coated, HA–EDA-coated polycarbonate membrane (*n* = 3). Data are expressed as average ± SEM. ** *p* < 0.01, ns = non-significant (*p* > 0.05) by one-way ANOVA with Tukey’s multiple comparison tests. Figure S2: DIC and immunofluorescence images of E12 and E13 eSMGs. CD44 (magenta), E-cadherin (green), and TUJ1 (para-sympathetic ganglion; red) are immunostained. Scale bar = 200 µm.
