# Supplementary material for: Efficient Surface Immobilization of Chemically Modified Hyaluronans for Enhanced Bioactivity and Survival of In Vitro-Cultured Embryonic Salivary Gland Mesenchymal Cells
Source: Polymers (Basel). 2021 Apr 9;13(8):1216. doi: 10.3390/polym13081216 (PMC8069509; doi:10.3390/polym13081216)
Supplement: Supplementary file 1 [file polymers-13-01216-s001.pdf]

polymers

MDPI

Supplementary

Efficient Surface Immobilization of Chemically Modified Hyaluronans for Enhanced Bioactivity and Survival of In vitro-Cultured Embryonic Salivary Gland Mesenchymal Cells

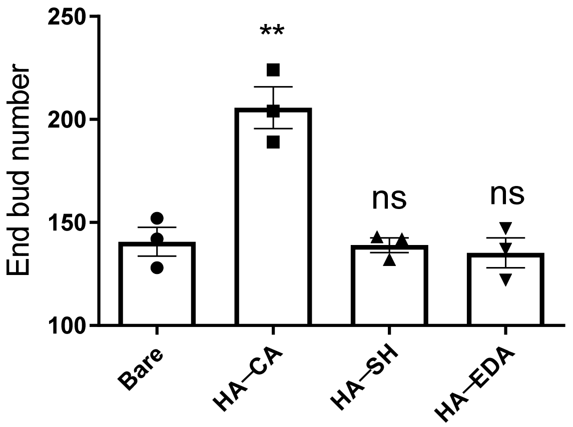

Figure S1. Quantification of bud number 24 h after eSMG culture on bare, HA–CA-coated, HA–SH-coated, HA–EDA-coated polycarbonate membrane (n = 3). Data are expressed as average ± SEM. **$p < 0.01$, ns = non-significant ($p > 0.05$) by one-way ANOVA with Tukey's multiple comparison tests.

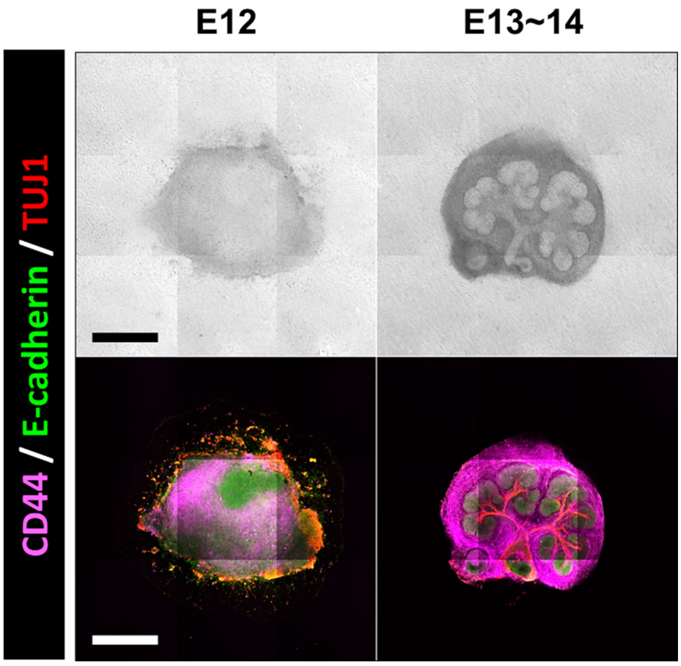

Figure S2. DIC and immunofluorescence images of E12 and E13 eSMGs. CD44 (magenta), E-cadherin (green), and TUJ1 (parasympathetic ganglion; red) are immunostained. Scale bar = 200 μm.
